# Nanoselenium application improves post‐harvest fruit quality and disease resistance in tomato

**DOI:** 10.1111/tpj.70432

**Published:** 2025-08-22

**Authors:** Xiaoqing He, Shiyu Ying, Yi Xu, Zhuo Gao, Yi Wu, Mingchun Liu, Mengbo Wu

**Affiliations:** ^1^ Key Laboratory of Bio‐Resource and Eco‐Environment of Ministry of Education, College of Life Sciences Sichuan University Chengdu 610065 Sichuan China; ^2^ Laboratoire de Recherche en Sciences Végétales—Génomique et Biotechnologie des Fruits—UMR5546 Université de Toulouse, CNRS, UPS, Toulouse‐INP Toulouse France; ^3^ Horticulture Institute Sichuan Academy of Agricultural Sciences Chengdu 610066 China

**Keywords:** nanoselenium, *Botrytis cinerea*, fruit quality, tomato

## Abstract

Fruits constitute a vital component of a nutritious diet but are highly perishable, contributing substantially to food waste. Consequently, identifying safe and edible biological agents to enhance product quality and extend shelf life is of critical importance. In this study, we demonstrate that exogenous pre‐harvest foliar application with nanoselenium (nano‐Se) in tomato enhances fruit quality, prolongs fruit shelf life, and enhances the resistance of tomato fruit to *Botrytis cinerea* infection. Transcriptomic analysis revealed coordinated upregulation of genes associated with quality maintenance and modulation of phytohormone‐related pathways. Notably, nano‐Se treatment induced expression patterns of ripening‐related genes that resembled those triggered by ethylene (ET) but were antagonistic to the effects of 1‐MCP. We further demonstrated that although *SlMYC2* knockout increased susceptibility to *B. cinerea*, nano‐Se application restored resistance in a manner independent of the SlMYC2‐associated jasmonic acid signaling pathway, implicating ET as the primary regulatory mechanism. Collectively, these findings support nano‐Se as a promising biostimulant for reducing post‐harvest losses while preserving the nutritional and sensory quality of tomato fruits.

## Significance Statement

This study demonstrates that nanoselenium treatment of tomato can significantly improve both fruit quality and disease resistance. These findings provide a novel theoretical foundation and technological approach for developing environmentally friendly preservatives.

## INTRODUCTION

The formation of fruit quality traits is a complex process, resulting in significant variability in color, texture, flavor, and nutritional composition (Zhu et al., 2022). Tomato (*Solanum lycopersicum*) is among the most widely consumed fruits and vegetables worldwide, valued for its high content of dietary fiber, vitamins, carotenoids, and antioxidants (Ali et al., 2020). However, tomato fruits undergo a climacteric ripening process characterized by a dramatic increase in ethylene (ET) production, which accelerates softening, reduces post‐harvest shelf life, and heightens susceptibility to fungal infections—ultimately causing substantial economic losses (Alexander, 2002; Arah et al., 2016; Changwal et al., 2021). Due to their high sensitivity to pathogens, tomato fruits often require extensive use of chemical disease control agents and insecticides during production and post‐harvest handling to combat threats such as *Botrytis cinerea*, powdery mildew, bruising, and blight (Borrelli et al., 2018; Ferrero et al., 2020). Over‐ripening and decay induced by pathogen infection are the principal contributors to post‐harvest losses, severely impairing the organoleptic quality of the fruit and rendering it unsuitable for consumption. Therefore, minimizing pesticide use while effectively controlling diseases and maintaining fruit quality remains a critical challenge in tomato production and post‐harvest processes (Damalas & Eleftherohorinos, 2011; Panno et al., 2021).

The nanoselenium (nano‐Se) plant health activator employs specific microbial strains to efficiently convert selenium into bioactive nano‐size particles ranging from 20 to 60 nm. These nanoparticles exhibit high bioavailability and are rapidly absorbed, utilized, and metabolized by both the plants and the human body, with significantly lower toxicity compared with inorganic and organic selenium forms (Li et al., 2020; Liu, Deng, et al., 2022; Liu, Huang, et al., 2022). Recent studies have begun to elucidate the role of nano‐Se in plant growth and development (Gudkov et al., 2020; Hussein et al., 2019; Samynathan et al., 2023; Shiriaev et al., 2023). For instance, in rice, nano‐Se treatment has been shown to enhance yield by improving photosynthetic efficiency (Badawy et al., 2021; Wang et al., 2021), while in wheat, selenium supplementation improves germination rate and seed vigor (Ghazi et al., 2022). Additionally, nano‐Se application has been associated with increased accumulation of bioactive metabolites, such as soluble sugars, phenols, flavanols, tocopherols, and anthocyanins in wheat (Sardari et al., 2022).

Nano‐Se also plays a pivotal role in the formation of fruit quality traits and the post‐harvest preservation of fruits and vegetables. In crops, such as pepper, tea, and strawberries, nano‐Se has been reported to promote secondary metabolism and the accumulation of antioxidant compounds (Huang, Yu, et al., 2023; Li et al., 2020; Shiriaev et al., 2023; Tran et al., 2023). Foliar application of nano‐Se on pepper plants enhanced the biosynthesis of capsaicin, brassinosteroids (BRs), and total phenolics, along with a significantly increased level of jasmonic acid (JA), abscisic acid (ABA), and salicylic acid (SA) levels (Li et al., 2020). In tea, nano‐Se reduced the levels of polyphenols, catechins, and caffeine, thereby decreasing bitterness and astringency while improving the umami and sweetness profile (Huang, Yu, et al., 2023). In strawberries, nano‐Se coating significantly increased fruit firmness, reduced water loss, and preserved approximately 50% of the original vitamin C and B_9_ content compared with untreated controls (Tran et al., 2023). More recently, Liu et al. developed a temperature‐responsive selenium nanoparticle gel (Ipr@MSe@LA NPs) that adheres to the surface of *B. cinerea*, facilitating its rapid capture and inactivation, thus markedly extending the strawberry shelf life (Liu, Huang, et al., 2022). In tomato, exogenous application of nano‐Se under pyrithione‐induced stress conditions increased soluble sugar and volatile compound contents while reducing organic acid accumulation (Liu, Deng, et al., 2022). Moreover, nano‐Se outperformed nano‐TiO_2_ and nano‐CeO_2_ in improving the flavor quality of tomato fruits (Liu et al., 2023). While several studies have demonstrated the positive effects of nano‐Se on tomato fruit quality, the underlying molecular mechanisms remain largely unexplored. More critically, it is still unclear whether nano‐Se can improve post‐harvest disease resistance in tomato and effectively extend shelf life.

This study demonstrates that foliar application of 20 mg L^−1^ nano‐Se significantly improves tomato fruit quality by increasing Brix value, while accelerating pigment accumulation during ripening. Nano‐Se treatment effectively delayed fruit dehydration and enhanced resistance to *B. cinerea* infection. Notably, the application of nano‐Se substantially elevated the level of γ‐aminobutyric acid (GABA), a key nutritional and bioactive compound. Transcriptomic analysis revealed significant upregulation of genes associated with fruit ripening and quality formation in nano‐Se‐treated fruits. Moreover, nano‐Se modulated the coordinated expression of genes involved in multiple phytohormone biosynthesis and signaling pathways. Collectively, these findings provide a theoretical basis for the application of nano‐Se to enhance fruit ripening and post‐harvest stress resistance, offering an eco‐friendly and sustainable alternative to conventional chemical pesticides in tomato production.

## RESULTS

### Nano‐Se20 accelerates the onset of tomato fruit ripening

**Figure 1:**
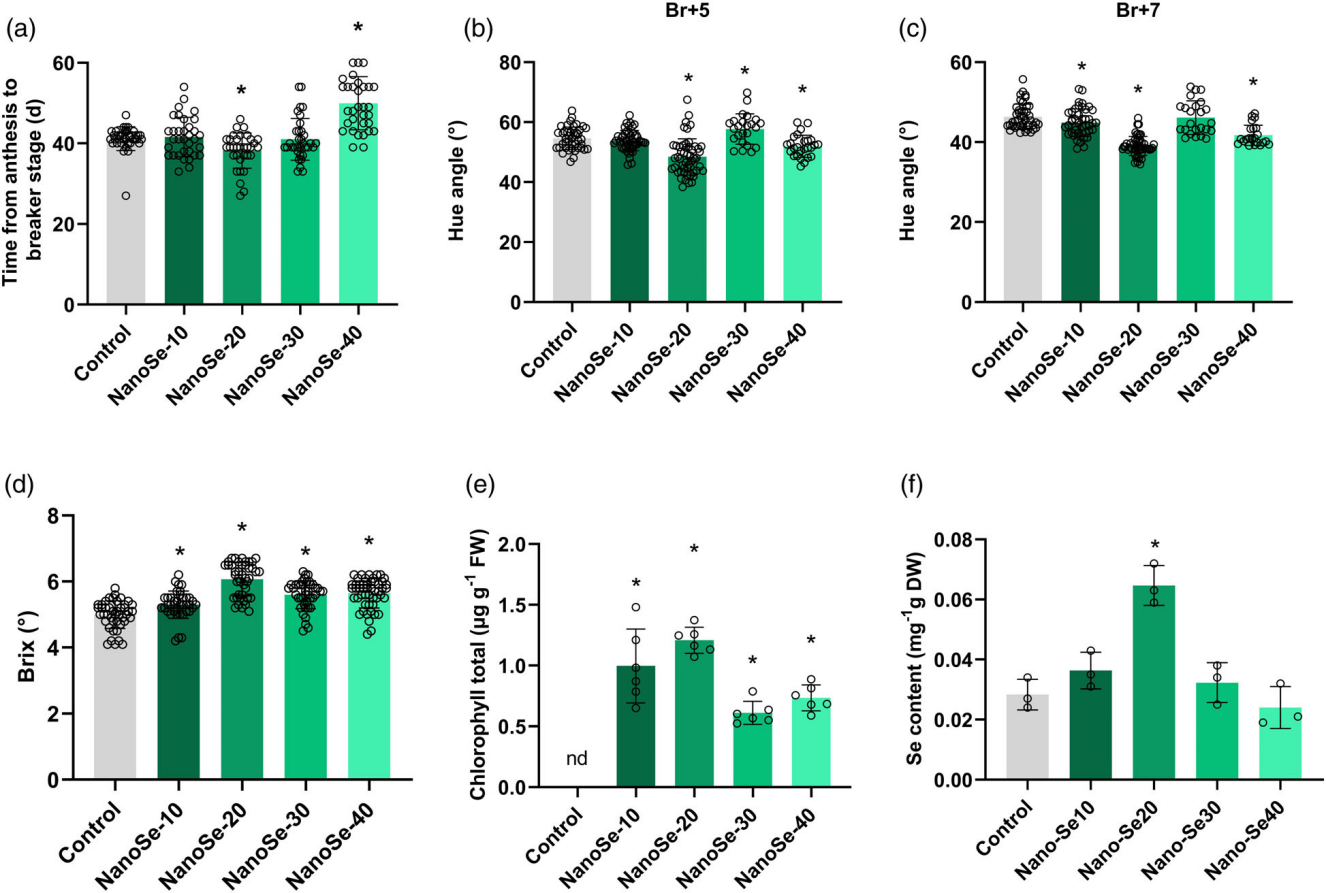
Nanoselenium accelerates the ripening process of tomato fruit. (a) Time from anthesis to breaker stage in control and nano‐Se10, 20, 30, 40 fruits. Data are shown as means ± standard deviation (SD). Each small circle represents a fruit. Asterisks indicate statistical significance using Student's *t*‐test, *P* < 0.05. (b, c) Hue angle changes of control and nano‐Se10, 20, 30, 40 fruits at different maturation stages. Data are shown as means ± standard deviation (SD). Each small circle represents a fruit. Br: Breaker, Br+1‐7 days after the Br stage. Asterisks indicate statistical significance using Student's *t*‐test, *P* < 0.05. (d) Tomato fruit Bailey sugar content of control and nano‐Se10, 20, 30, 40 fruits at Br+7 stage. Data are shown as means ± standard deviation (SD). Each small circle represents a fruit. Asterisks indicate statistical significance using Student's *t*‐test, *P* < 0.05. (e) Chlorophyll content of tomato in control and nano‐Se10, 20, 30, 40 fruits at Br+7 stage. Data are shown as means ± standard deviation (SD). Each small circle represents a biological replicate. Asterisks indicate statistical significance using Student's *t*‐test, *P* < 0.05. (f) Se content in tomato after foliar application of different concentrations of nano‐Se. Asterisks indicate statistical significance using Student's *t*‐test, *P* < 0.05.

To comprehensively elucidate the role of nano‐Se in tomato fruit ripening, we assessed the effect of pre‐harvest foliar application with different nano‐Se concentrations on the onset of ripening. The time interval from anthesis to the breaker stage was recorded in tomato plants treated with various concentrations of nano‐Se. Results showed that 10 and 30 mg L^−1^ treatments have no significant effect on ripening initiation compared with the control, while 20 mg L^−1^ nano‐Se significantly accelerated the ripening onset (Figure 1a). In contrast, the 40 mg L^−1^ treatment delayed the breaker stage (Figure 1a). To further evaluate the impact of nano‐Se on the ripening process, hue angle measurements were conducted at different ripening stages (Br+5 and Br+7) across all treatment groups. The data revealed that nano‐Se‐treated fruits generally exhibited more rapid pigment accumulation than the control, with the exception of 10 mg L^−1^ at Br+5 and 30 mg L^−1^ at Br+7 stages (Figure 1b,c). At the Br+7 stage, the Brix sugar content was significantly higher in fruits treated with nano‐Se than in control fruits (Figure 1d). In the Br+7 stage, the chlorophyll content in nano‐Se‐treated fruits increased significantly (Figure 1e), which was consistent with the elevated sugar content (Luo et al., 2013). Given that nano‐Se is efficiently absorbed by the human body (Jiang et al., 2017) and serves as an indicator of tomato nutritional quality, selenium content was qualified across all treatments. The highest selenium accumulation was detected in fruits treated with 20 mg L^−1^ nano‐Se (Figure 1f). Taken together, the results indicate that 20 mg L^−1^ is the optimal concentration for improving tomato fruit quality, as it promotes earlier ripening, enhances pigment and sugar accumulation, and enriches the fruit with bioavailable selenium.

### Nano‐Se20 enhances tomato fruit firmness and reduces post‐harvest water loss

**Figure 2:**
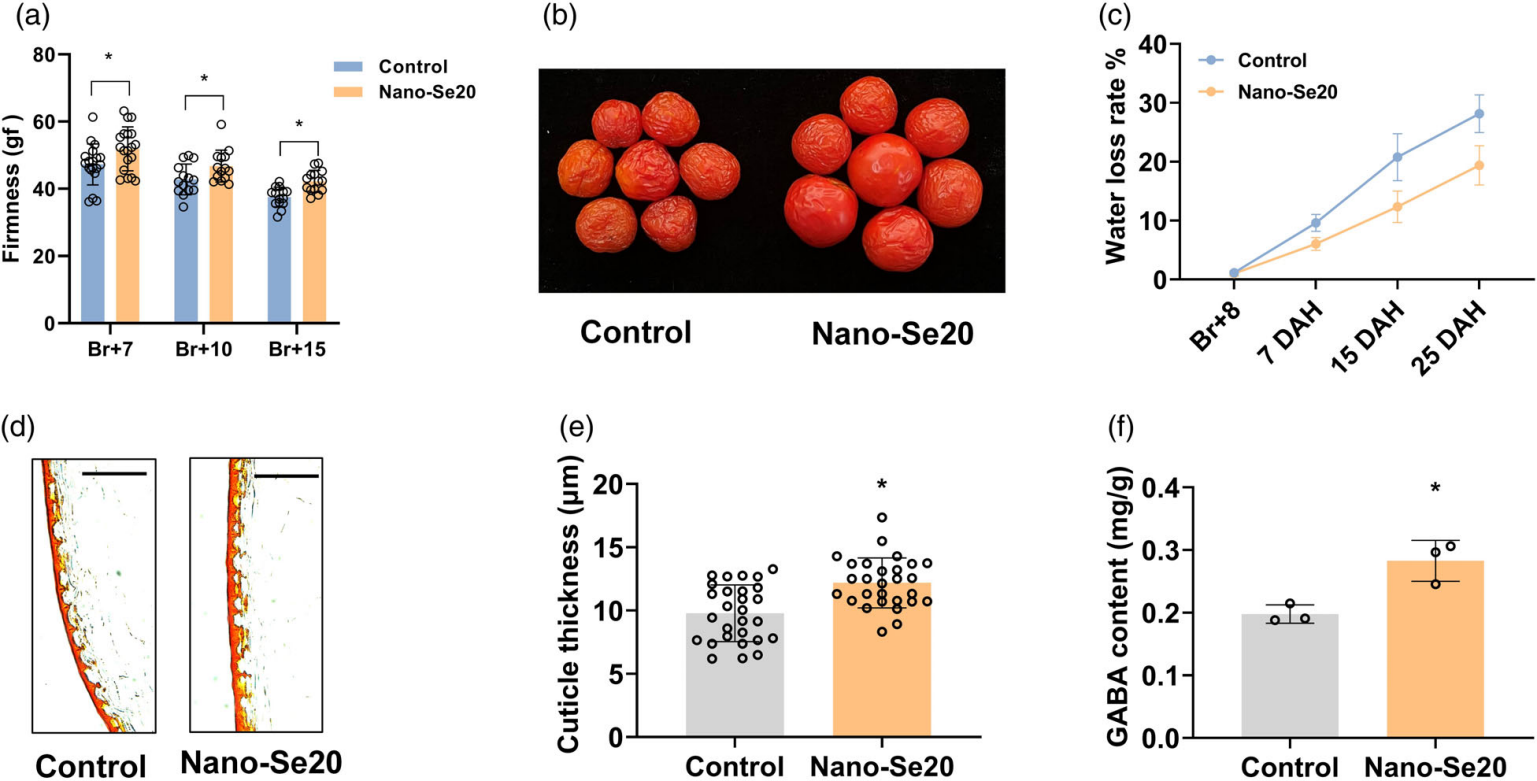
Nano‐Se20 increases tomato fruit firmness and extends shelf life. (a) Firmness of control and nano‐Se20 fruits at the Br+7, Br+10, and Br+15 stages; Br: Breaker. Data are shown as means ± standard deviation (SD). Each small circle represents a fruit. Asterisks indicate statistical significance using Student's *t*‐test, *P* < 0.05. (b) Control and nano‐Se20 fruits harvested at Br+7 stage. Photographs were taken at 25 days of storage. (c) Control and nano‐Se20 fruits harvested at Br+7 stage; water loss rate of control and nano‐Se20 fruits was measured every week. DAH, days after harvested. (d) Cuticle thickness of control and nano‐Se20 fruits at Br+7. (e) Cuticle thickness measurements were performed on Br+7 control and nano‐Se20 fruits. Data are shown as means ± standard deviation (SD). Asterisks indicate statistical significance using Student's *t*‐test, *P* < 0.05. (f) The GABA content in control and nano‐Se20‐treated fruits at the Br+7 stage was determined from three biological replicates. Asterisks indicate statistical significance using Student's *t*‐test, *P* < 0.05.

Given the previously demonstrated role of 20 mg L^−1^ nano‐Se in promoting tomato fruit ripening (Figure 1a), and recognizing that post‐harvest shelf life is a critical trait for marketability, we further evaluated the impact of nano‐Se20 on fruit firmness and water loss ratio. Fruit firmness was measured at Br+7, Br+10, and Br+15 stages in both control and treated groups. Results showed that nano‐Se20‐treated fruits consistently exhibited significantly higher firmness across all time points compared with the control (Figure 2a), indicating improved textural integrity during ripening and post‐harvest storage. To assess post‐harvest dehydration, fruits were harvested at the Br+7 stage and stored under ambient conditions. Water loss was monitored by recording fruit weight at 1, 7, 15, and 25 days after harvesting. Nano‐Se20 treated fruits demonstrated a slower rate of water loss compared with the control (Figure 2b,c), suggesting enhanced water retention capacity. Oil Red O staining of the fruit pericarp at the Br+7 stage revealed that there was a noticeably thicker cuticle layer in nano‐Se20 treated fruits relative to controls (Figure 2d,e), which likely contributed to the change of cutin metabolism. In addition to structural preservation, nutritional enhancement was also observed. GABA, a non‐protein amino acid with known health‐promoting properties (Heli et al., 2022), is typically upregulated during late ripening and post‐harvest stages. Quantification of GABA levels revealed a substantial increase in nano‐Se20 treated fruit compared with the control group (Figure 2f), indicating a potential nutritional benefit associated with treatment. In conclusion, application of 20 mg L^−1^ nano‐Se enhances tomato fruit firmness, reduces post‐harvest water loss, and increases GABA accumulation, thereby contributing to extended shelf life and improved nutritional quality.

### Nano‐Se20 treatment improves tomato fruit tolerance to *B. cinerea*

**Figure 3:**
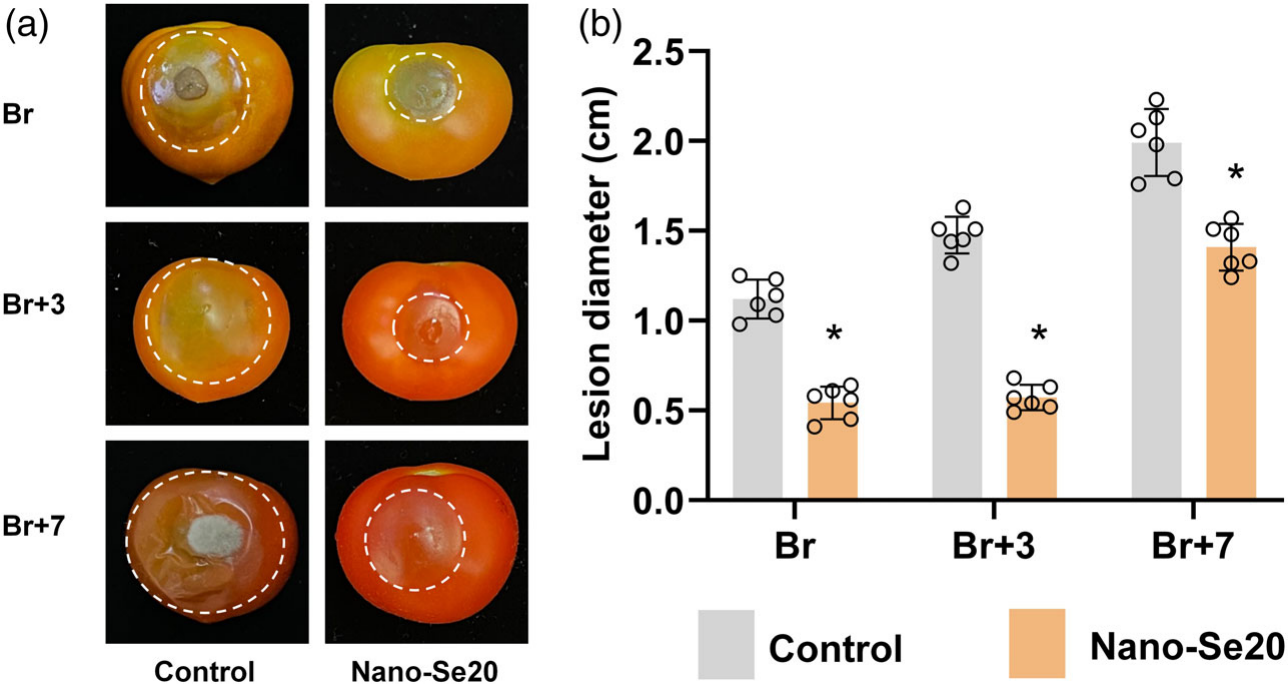
Nano‐Se20 improves disease resistance in tomato fruits against *Botrytis cinerea* infection. (a) Br, Br+3, and Br+7 stage control and nano‐Se20 fruits were inoculated with *B. cinerea* and photographed after 72 h of treatment. (b) Lesion diameters of Br, Br+3, and Br+7 stage control and nano‐Se20 fruits were inoculated with nano‐Se20 and photographed after 72 h of treatment. Means were calculated from six individual fruits. Data are shown as means ± standard deviation (SD). Each small circle represents a fruit. Asterisks indicate statistical significance using Student's *t*‐test; *P* < 0.05.

To address the impact of nano‐Se20 on tomato fruit resistance to *B. cinerea* infection, fruits at different ripening stages (Br, Br+3, and Br+7) were inoculated with the pathogen. Lesion diameters were measured 72 h post‐inoculation. Compared with the control, nano‐Se20‐treated fruits exhibited significantly smaller lesion areas across all ripening stages (Figure 3a,b). Notably, the degree of infection increased with fruit ripening, as lesion diameters averaged approximately 0.5 cm at both Br and Br+3 stages, and 1.5 cm at Br+7 (Figure 3b). These results indicate that nano‐Se20 treatment enhances tomato fruit resistance to *B. cinerea*.

### Upregulation of quality‐ and disease resistance‐related genes in nano‐Se20 treated tomato fruits

**Figure 4:**
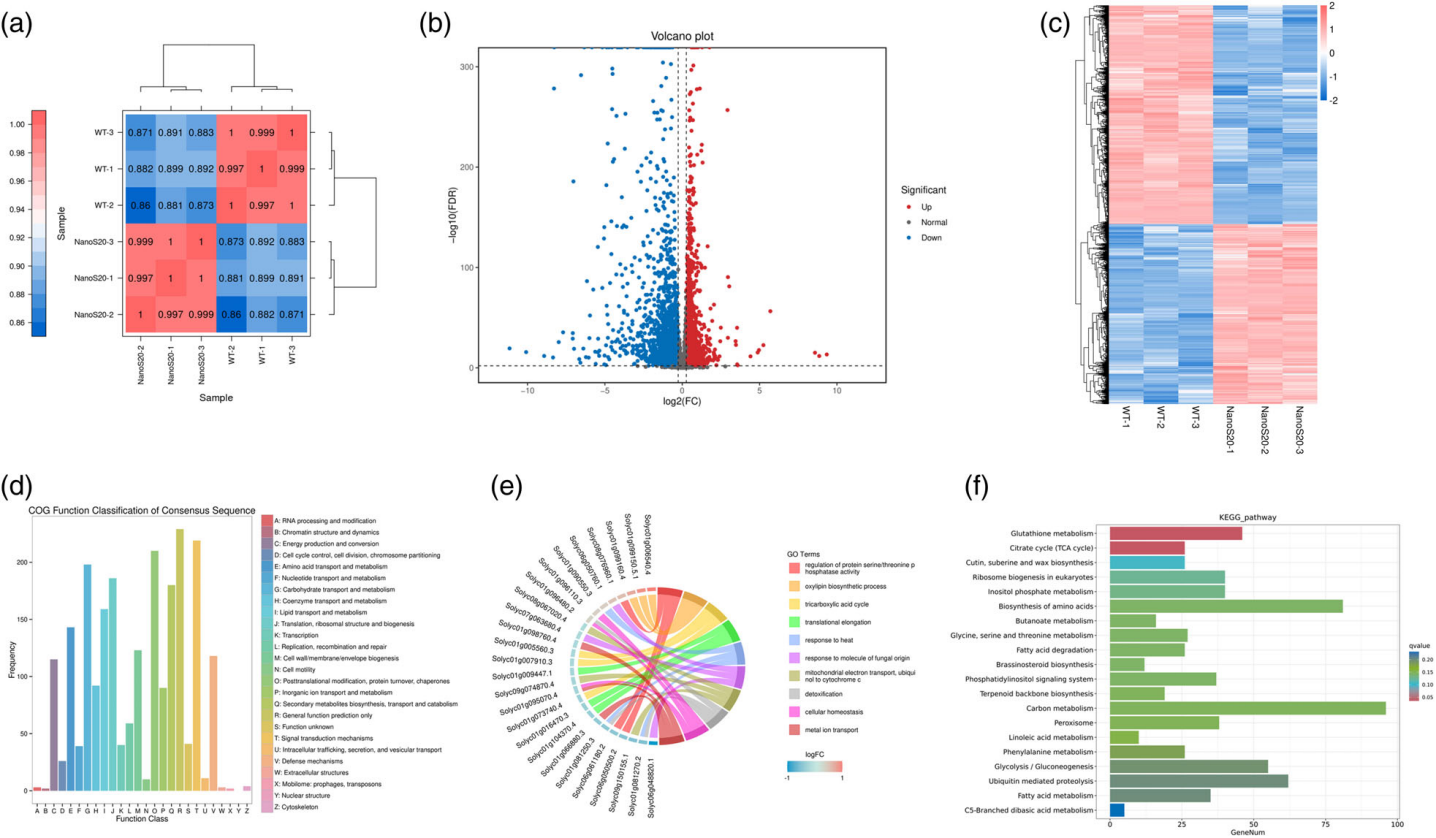
RNA‐seq profiling of control and nano‐Se20 fruits. (a) The Pearson correlation coefficient (*r*) serves as an evaluative metric for assessing the correlation between biological replicates. The closer the computed *r* ^2^ value is to 1, the stronger the correlation between the two replicate samples. (b) Volcano plot displaying the differential gene expression profiles in control and nano‐Se20 Br+7 fruits. (c) Hierarchical clustering analysis was performed on all identified differentially expressed genes (DEGs) to group genes with identical or similar expression patterns across different samples, which were subsequently visualized using a heatmap. (d) Statistical results of Clusters of Orthologous Groups classification for DEGs. (e) Kyoto Encyclopedia of Genes and Genomes (KEGG) annotation of DEGs between control and nano‐Se20 Br+7 fruits. Pathway significance enrichment analysis enables the identification of the predominant biochemical metabolic pathways and signal transduction pathways associated with the candidate genes. The enrichment results were visualized using bar plots via the Cluster Profiler package. (f) Gene ontology (GO) enrichment chord plot of DEGs. On the outer ring, the left side represents the DEGs, where the color intensity of gene blocks indicates the magnitude of fold change (red for upregulated genes, blue for downregulated genes). The right side of the outer ring displays GO terms, with distinct colors representing different functional categories. The connecting lines between genes and GO terms denote their enrichment relationships.

To elucidate the molecular basis of nano‐Se‐mediated regulation of fruit ripening and disease resistance, we conducted transcriptome sequencing on ripening tomato fruits (Br+7 stage) treated with 20 mg L^−1^ nano‐Se (NanoS20) and control fruits (wild‐type, WT) (Table S1). High reproducibility among three biological replicates in both groups (WT and NanoS20) was confirmed by Pearson's correlation coefficient (Figure 4a), ensuring the reliability of the transcriptomic data. Comparative analysis identified a total of 5506 differentially expressed genes (DEGs) between nano‐Se20‐treated and WT fruits, with 2486 genes upregulated and 3020 genes downregulated in the treated group (Figure 4b; Table S2). Hierarchical clustering of DEGs revealed distinct expression patterns between WT and NanoS20 samples, highlighting global transcriptional reprogramming induced by the application of nano‐Se (Figure 4c). Functional categorization using the Cluster of Orthologous Groups database showed DEGs were significantly enriched in categories related to cell wall biogenesis, secondary metabolite biosynthesis, and defense mechanisms—traits consistent with the enhanced fruit firmness and disease resistance observed phenotypically (Figure 4d). Gene ontology enrichment analysis further indicated that DEGs were associated with biological processes such as response to fungus elicitors and adaptation to heat stress (Figure 4e). Pathway enrichment using the Kyoto Encyclopedia of Genes and Genomes database revealed that DEGs were predominantly involved in metabolic pathways including phenylpropanoid biosynthesis, cutin and wax biosynthesis, fatty acid metabolism, the tricarboxylic acid cycle, and BR biosynthesis (Figure 4f). These findings indicate that nano‐Se plays critical roles in structural integrity, secondary metabolism, and plant defense, suggesting that nano‐Se treatment enhances cuticular development and activates key stress‐responsive networks in tomato fruits.

**Figure 5:**
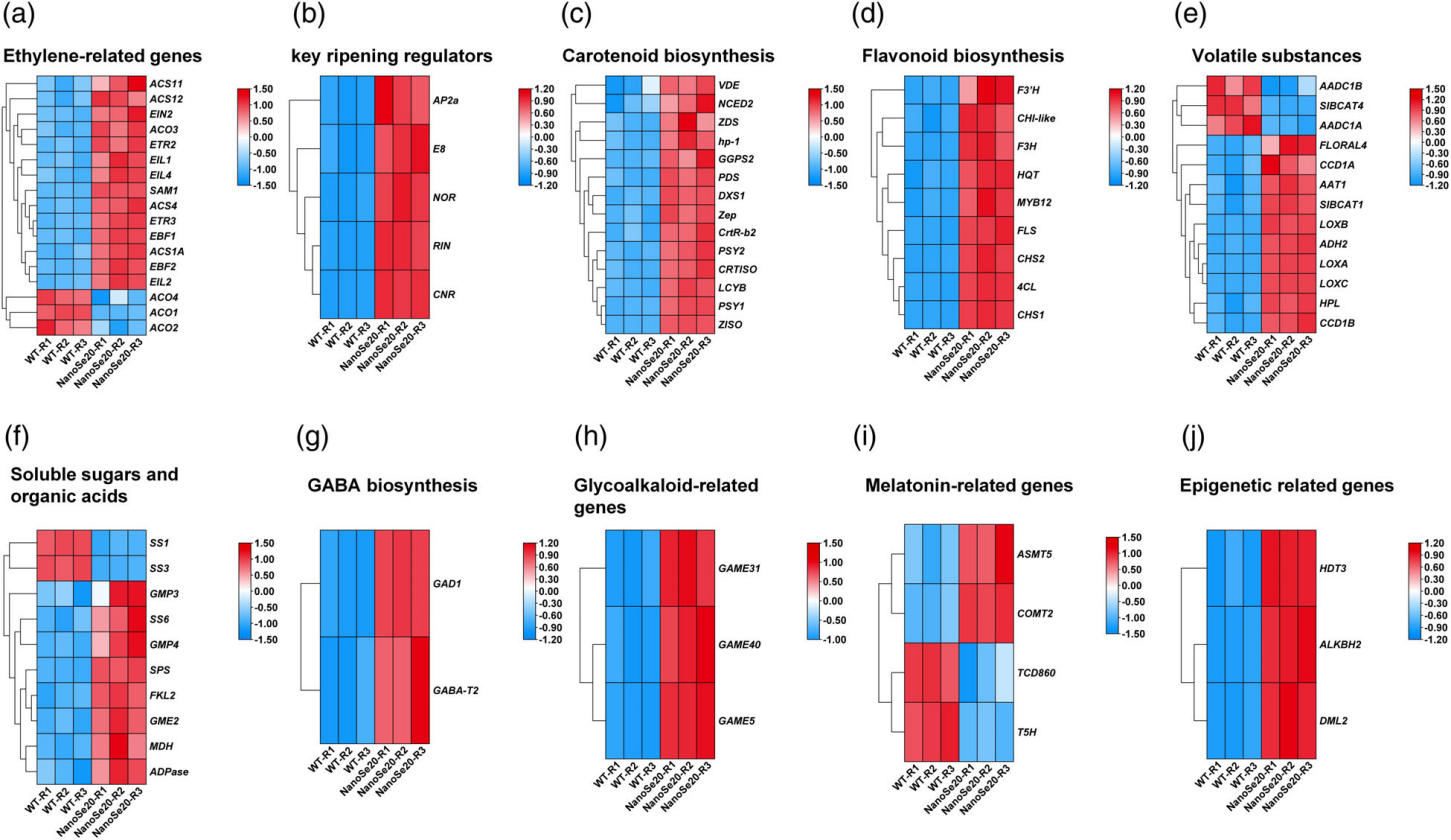
Heatmaps display the effects of nanose‐20 treatment on differentially expressed genes associated with ripening and quality in tomato fruits. (a) Ethylene biosynthesis‐related genes. (b) Key transcription factors regulating fruit ripening. (c) Carotenoid biosynthesis‐related genes. (d) Flavonoid biosynthesis‐related genes. (e) Volatile aroma compound‐related genes. (f) Soluble sugar and organic acid‐related genes. (g) GABA‐related genes. (h) Ripening‐specific glycoalkaloid‐related genes. (i) Melatonin‐related genes. (j) Epigenetic regulation‐related genes.

Comparative transcriptomic analysis revealed significant upregulation of key genes associated with fruit ripening and quality in nano‐Se20‐treated fruits compared with WT controls. ET biosynthesis and signaling genes—including *ACS4*, *ACO3*, *EIL1*, *EIL2*, and *EIL4*—were upregulated, whereas *ACO1* expression was significantly reduced (Figure 5a). Key ripening regulators, such as *RIN*, *NOR*, and *CNR* also showed consistent upregulation, indicating that nano‐Se activated a central transcriptional network to promote ripening (Figure 5b). Genes involved in carotenoid biosynthesis—including *PSY1*, *PDS*, *ZDS*, *ZISO*, *CRTISO*, and *LCYB—*were coordinately upregulated (Figure 5c), as were those responsible for flavonoid biosynthesis and regulation, such as *CHS1*, *CHS2*, *F3H*, *FLS*, *CHIL1*, *CHIL2*, *F3'H*, and *MYB12* (Figure 5d), suggesting enhanced phytonutrient production in treated fruits. Additionally, genes involved in volatile aroma compound and soluble sugar metabolism—including *ADH2*, *LoxA*, *LoxB*, *LoxC*, *CCD1B*, *HPL*, *AAT1*, and *BCAT1* for volatiles; and *SS6*, sucrose phosphate synthase (*SPS*), and *FKL2* for sugars—were significantly upregulated (Figure 5e,f). Upregulation of *GMP3* and *MDH*, associated with organic acid biosynthesis genes, suggests nano‐Se also modulates sugar–acid balance (Figure 5f). Intriguingly, GABA synthesis genes *GAD1* and *GABA*‐*T2* were upregulated, offering a mechanistic basis for the observed accumulation of GABA (Figure 5g). Unexpectedly, fruit‐specific glycoalkaloid biosynthesis genes *GAME31*, *GAME40*, and *GAME5* were also upregulated. Although these compounds are generally associated with bitterness, their accumulation may contribute to enhanced disease resistance and extended storage life (Bai et al., 2024) (Figure 5h). Similarly, melatonin synthesis genes *ASMT5* and *COMT2* were induced, likely improving both nutritional value and stress tolerance (Zhang et al., 2023) (Figure 5i). Interestingly, epigenetic regulators *DML2*, *HDT3*, and *ALKBH2—*which are known to delay ripening when overexpressed—were also upregulated, suggesting a possible role of nano‐Se in modulating fruit ripening and shelf life via epigenetic mechanisms (Figure 5j). These findings demonstrate a dual functionality of nano‐Se: (1) activation of ripening and quality‐associated genes to improve flavor and nutritional traits, and (2) stimulation of antioxidant and defense‐related pathways that support post‐harvest performance.

**Figure 6:**
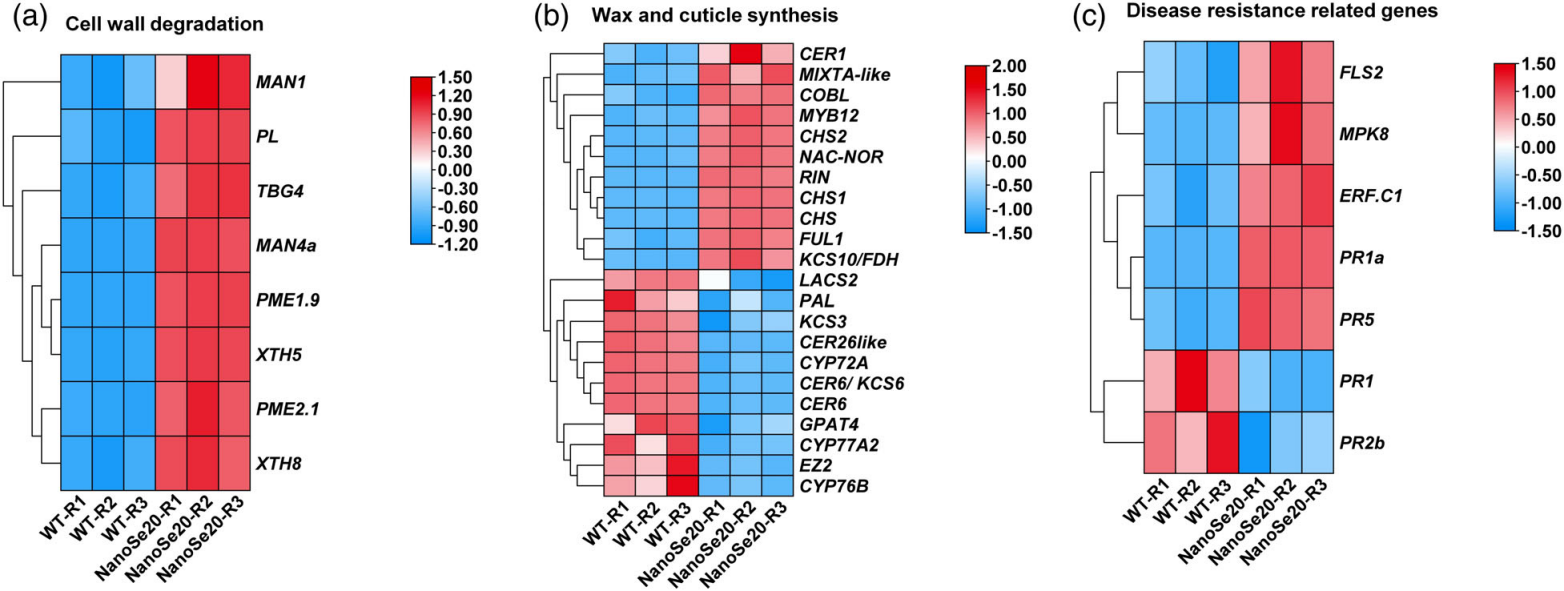
Heatmaps illustrating the effects of nanose‐20 treatment on differentially expressed genes related to cell wall degradation, disease resistance, wax, and cutin in tomato fruits. (a) Cell wall‐related genes. (b) Wax and cutin‐related genes. (c) Disease resistance‐related genes.

Maintaining fruit firmness, cuticle and wax content, and resistance to fungal pathogens is essential for post‐harvest quality. Despite the upregulation of cell wall‐softening‐related genes such as *PL*, *TBG4*, *XTH5*, *XTH8*, *PME1*.9, and *PME2.1* (Figure 6a), nano‐Se20‐treated fruits exhibited increased fruit firmness. This apparent paradox suggests that improved fruit hardness may result not from cell wall degradation but rather from increased accumulation of secondary metabolites, such as glycoalkaloids, melatonin, or structural reinforcements such as cuticular waxes. Supporting this hypothesis, genes involved in wax and cutin biosynthesis—including *CER1*, *MIXTA*‐like, *KCS10*/*FDH*, and *COBL*—as well as their regulatory transcription factors (*RIN*, *NOR*, *MYB12*) were significantly upregulated (Figure 6b), indicating that nano‐Se enhanced cuticle development and extended shelf life.

To assess fungal resistance, we focused on genes responsive to *B. cinerea*, a major fungal pathogen affecting post‐harvest tomato quality, which triggers significant economic losses. Nano‐Se treatment resulted in strong upregulation of pathogen‐responsive defense genes (*PR1a*, *PR5*, and *FLS2*) and ET‐mediated resistance genes (*ERF*.*C1*and *MAPK8*) (Figure 6c), reinforcing the idea that nano‐Se activates immune defense mechanisms to reduce fungal damage.

**Figure 7:**
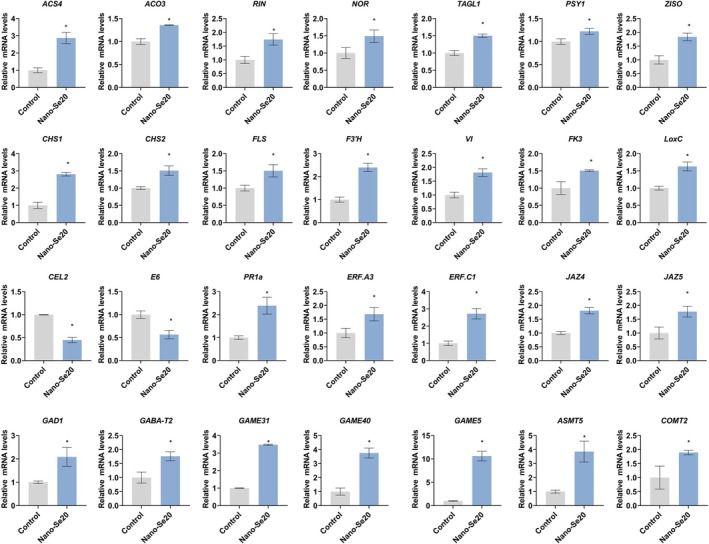
RT‐qPCR analysis of quality and disease resistance genes in control and nano‐Se20 fruits. Each value represents the mean of three biological replicates. Data are shown as means ± standard deviation (SD). Asterisks indicate statistical significance using Student's *t*‐test; *P* < 0.05.

Finally, reverse transcriptase‐quantitative polymerase chain reaction (RT‐qPCR) validation confirmed the upregulation of key genes involved in fruit ripening, quality maintenance, and defense responses in nano‐Se20‐treated fruits (Figure 7). Collectively, these data demonstrated that nano‐Se treatment reprograms gene expression to enhance both the internal quality and post‐harvest resistance of tomato fruit.

### Nano‐Se treatment orchestrates a multilayered regulatory network to remodel phytohormone signaling in tomato fruit

**Figure 8:**
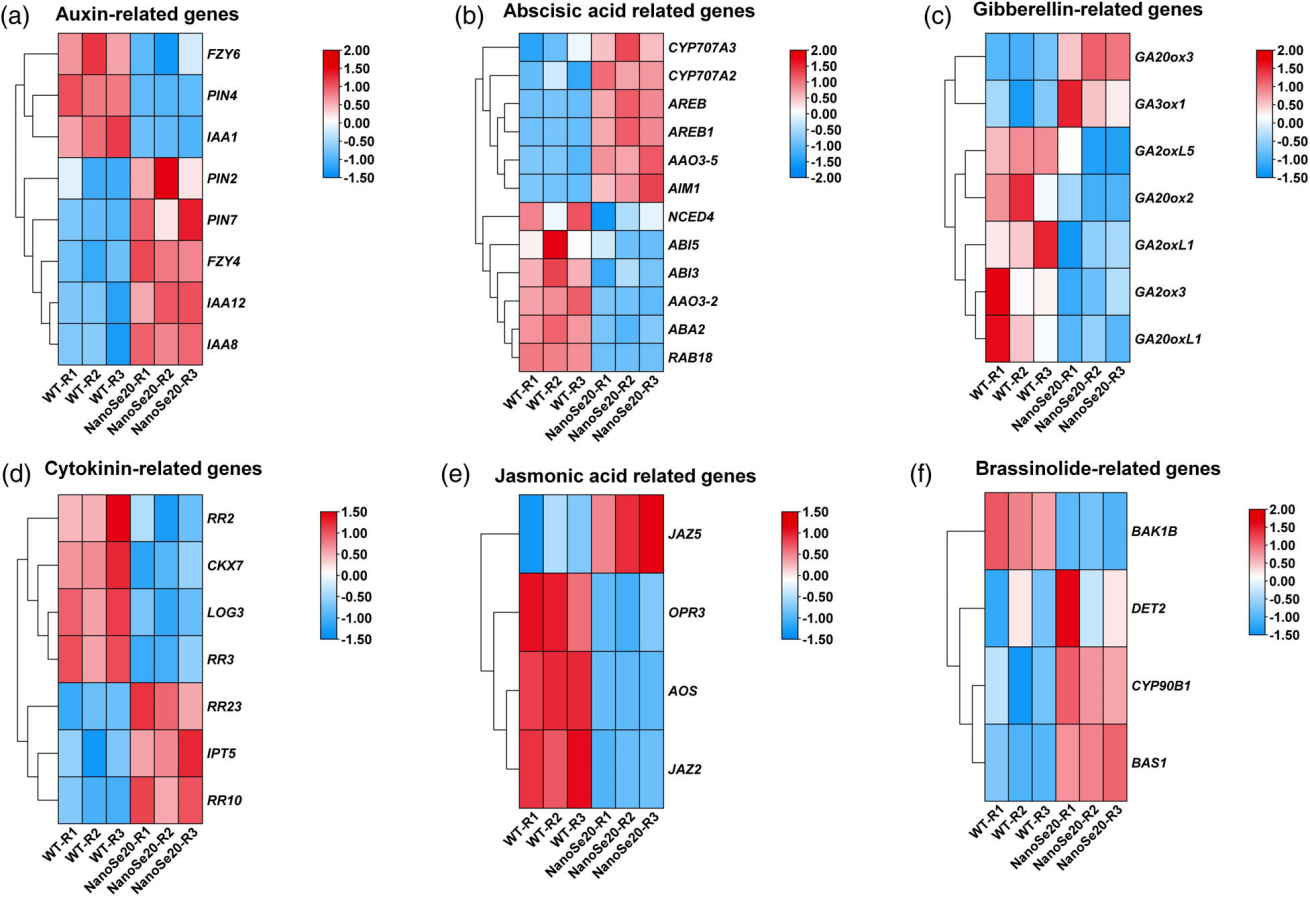
Heatmap showing the impact of nanoSe‐20 treatment on the expression of hormone‐related genes. The overview of main hormone DEGs related to synthesis, degradation, and signaling for auxin (a), abscisic acid (b), gibberellin (c), cytokinin (d), jasmonic acid (d), and brassinosteroid (f).

Key auxin signaling genes—including *PIN2*, *PIN7*, *IAA8*, and *IAA12*—were significantly upregulated in nano‐Se treated fruits, potentially promoting fruit development and facilitating the transition to ripening onset (Figure 8a). Interestingly, nano‐Se exerted a bidirectional regulatory effect on ABA metabolism, simultaneously activating biosynthesis genes (*CYP707A3*, *CYP707A2*, *AREB*, *AREB1*, *AAO3‐5*, and *AIM1*) while simultaneously downregulating ABA response genes (*ABI5* and *ABI3*) (Figure 8b). This suggests a refined hormonal balance, fine‐tuning ABA signaling to support both stress response and ripening progression. Consistent with the natural hormonal shifts during maturation, nano‐Se treatment also led to downregulation of gibberellin (GA) and cytokinin (CK) biosynthetic genes, reflecting a physiological decline of these growth‐promoting hormones as ripening proceeds (Figure 8c,d). In the context of stress response pathways, nano‐Se induces reprogramming of JA and BR signaling. JA‐responsive genes were suppressed, while BR‐associated genes were notably upregulated (Figure 8e,f). This shift may present a strategic reallocation of signaling resources from defense‐oriented responses to pathways favoring fruit growth and development. However, most SA‐related genes were not identified as differentially expressed, except for *PR1a* and *PR5* (Figure 6c), suggesting that nano‐Se may be partially involved in the regulatory process of SA biosynthesis. Collectively, these findings highlight nano‐Se's capacity to precisely and hierarchically reconfigure phytohormone networks: enhancing proripening signals (auxin and BR), modulating stress‐responsive hormones (ABA), and attenuating growth‐promoting signals (GA and CK) and JA‐mediated defense responses. This integrated hormonal remodeling is associated with the synergistic improvement of both fruit quality and post‐harvest stress resistance, establishing nano‐Se as a potent modulator of hormone‐mediated developmental transitions in tomato fruit.

### Nano‐Se modulates tomato fruit ripening and quality through coordinated ET signaling pathway regulation

**Figure 9:**
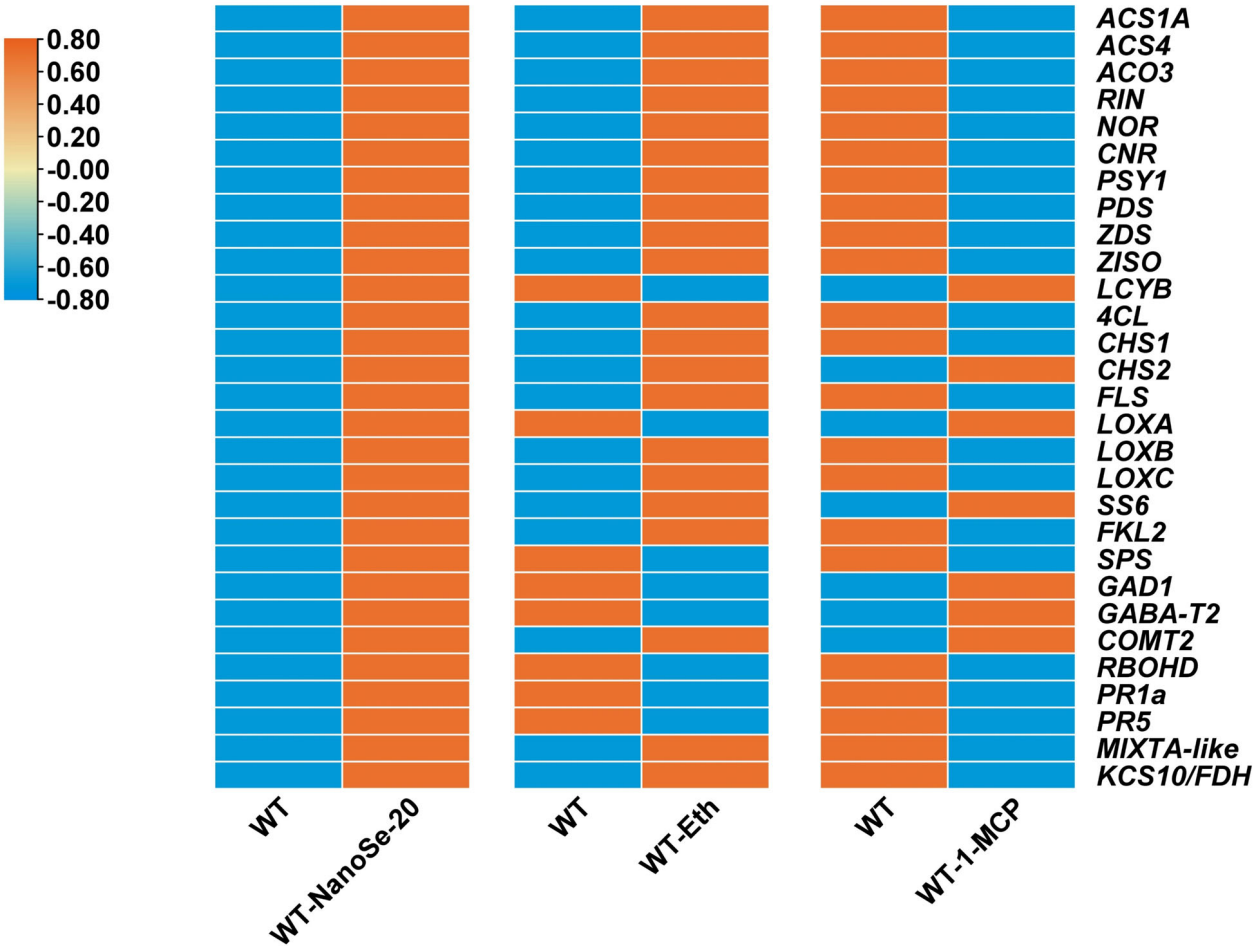
Heatmaps illustrate the effects of ethylene (ET) and the ET inhibitor 1‐MCP on Nanose‐20‐induced upregulation of ripening‐ and quality‐related genes in tomato fruits. Orange indicates upregulated expression, while blue represents downregulated expression. ET treatment was applied at the MG (mature green) stage, whereas the ET inhibitor (1‐MCP) was administered at the Br (breaker) stage.

ET and JA are key phytohormones that play distinct yet interconnected roles during tomato ripening processes—ET promotes ripening, while JA supports defense responses generally (Yang et al., 2024). To uncover the regulatory mechanisms of nano‐Se during this process, we compared the expression profiles of key ripening‐related genes under nano‐Se (nano‐Se20), ET (Eth), and ET inhibitor (1‐MCP) treatments. Our results show that nano‐Se treatment largely mimics the effects of ET, significantly upregulating ET biosynthesis genes (*ACS1A*, *ACS4*, and *ACO3*), master ripening regulators (*RIN*, *NOR*, and *CNR*), and quality‐associated genes involved in carotenoid (*PSY1*, *PDS*, *ZSD*, and *ZISO*), flavonoid (*4CL*, *CHS1*, *CHS2*, and *FLS*), volatile aroma (*LoxB* and LoxC) and cuticle biosynthesis (*MIXTA*‐like and *KCS10*/*FDH*) pathways. In contrast, these genes were strongly repressed under 1‐MCP treatment (Figure 9), confirming their ET dependency. Notably, nano‐Se exhibits unique regulatory characteristics: (1) it upregulates β‐carotene biosynthesis gene (*LCYB*), volatile compound gene (*LoxA*), and GABA synthesis genes (*GAD1* and *GABA‐T2*)—all of which are conversely suppressed by ET treatment; (2) the soluble sugar synthesis gene (*SS6*) and melatonin biosynthesis gene (*COMT2*) are consistently upregulated under all three treatments (Figure 9). Furthermore, nano‐Se specifically induces *SPS* and defense‐related genes (*RBOHD*, *PR1a*, and *PR5*), which are notably downregulated by ET treatment (Figure 9). These results suggest that nano‐Se enhances tomato fruit ripening and quality formation primarily via ET‐dependent signaling, yet also activates distinct metabolic and defense‐related pathways independently of ET. This dual‐mode regulation enables nano‐Se to improve both flavor attributes and post‐harvest disease resistance, positioning it as a versatile modulator of fruit quality and shelf life. It is noteworthy that while ET appears to be the primary regulator mediating nano‐Se‐induced quality improvement, shelf life extension, and disease resistance, the potential synergistic effects of other phytohormones warrant consideration.

### Nano‐Se20 enhances tomato resistance to *B. cinerea* partially independent of the SlMYC2‐mediated pathway

**Figure 10:**
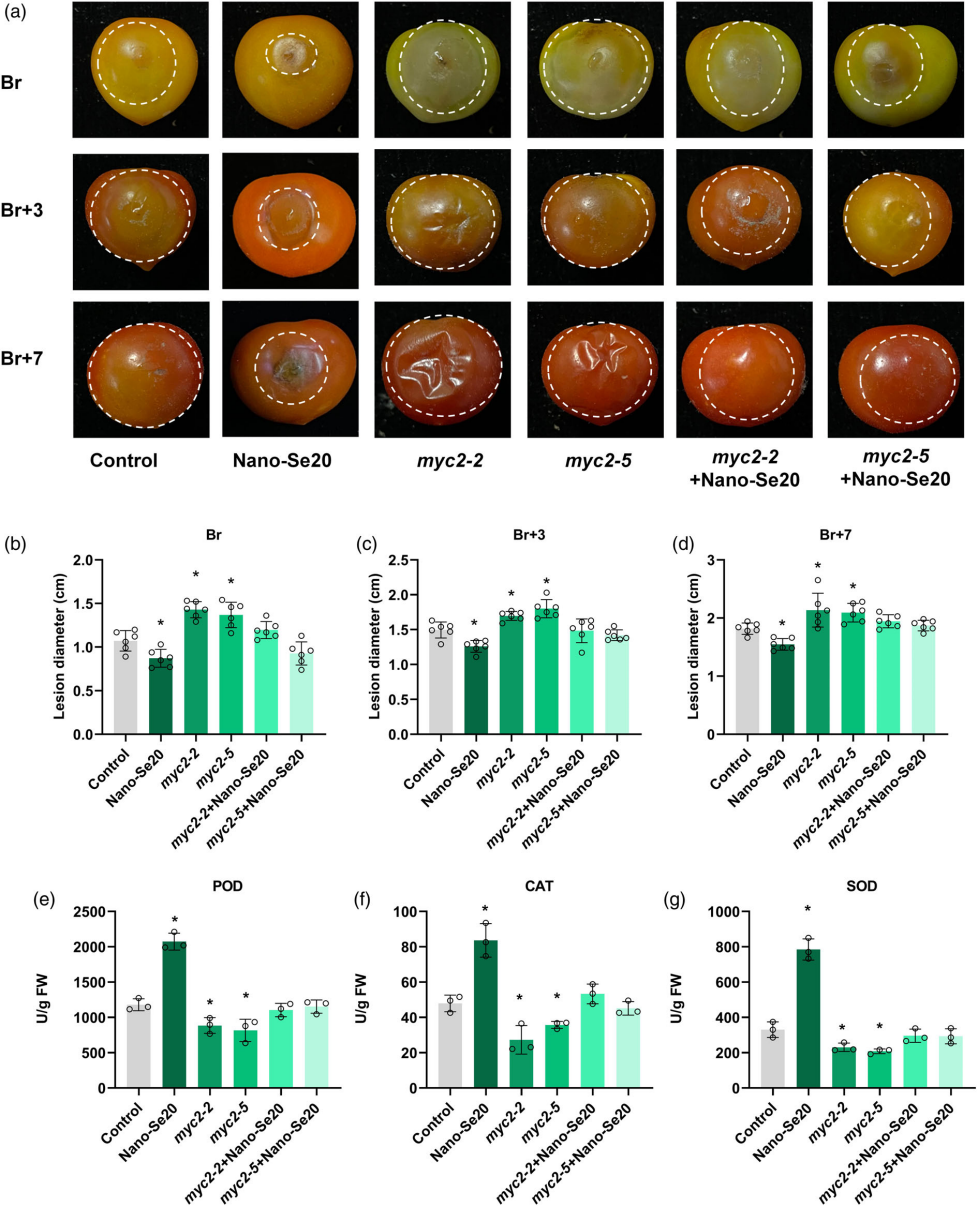
Nano‐Se20 improves disease resistance in tomato fruits. (a) Control, nano‐Se20, *myc2‐5*, *myc2‐7*, *myc2‐5+*nano‐Se20, *myc2‐7+*nano‐Se20. Br, Br+3 and Br+7 stage fruits were inoculated with nano‐Se20 and photographed after 72 h of treatment. (b–d) Lesion diameters of control, nano‐Se20, *myc2‐5*, *myc2‐7*, *myc2‐5*+nano‐Se20, *myc2‐7*+nano‐Se20 fruits after 72 h of inoculation with *Botrytis cinerea*. B is the Br stage; C is the Br+3 stage; and D is the Br+7 stage. Means were calculated from six individual fruits. Data are shown as means ± standard deviation (SD). Asterisks indicate statistical significance using Student's *t*‐test, *P* < 0.05. (e–g) Antioxidant enzymatic activities of POD, CAT, and SOD after inoculation with *B. cinerea*. Each value represents the mean of three biological replicates. Data are shown as means ± standard deviation (SD). Asterisks indicate statistical significance using Student's *t*‐test, *P* < 0.05.

JA is a key defense hormone against necrotrophic pathogens like *B. cinerea*, with SlMYC2 acting as a central transcriptional regulator in JA signaling (Shu et al., 2020; Zhang et al., 2017). To determine whether nano‐Se (nano‐Se20) confers resistance to *B. cinerea* via the JA pathway, we generated SlMYC2 knockout lines (*myc2‐5* and *myc2‐7*) using CRISPR/Cas9 technology (Deng et al., 2024). Fruits at different ripening stages (Br, Br+3, and Br+7) from WT and *myc2* mutants were treated with either a mock solution (H_2_O) or nano‐Se20, followed by *B. cinerea* inoculation. Lesion development was monitored 72 h post‐infection. As expected, nano‐Se20 significantly reduced the lesion sizes at all ripening stages in WT fruits; *myc2* mutants exhibited larger lesions compared with WT, confirming SIMYC2's role in resistance (Figure 10a). However, nano‐Se20‐treated *myc2* mutants showed a partial restoration of resistance (Figure 10a), evidenced by a significant reduction in lesion size compared with mock‐treated mutants (Figure 10b–d). To further understand the mechanism, we assessed antioxidant enzyme activities. Nano‐Se20–treated fruits showed elevated levels of *POD*, *CAT*, and *SOD* compared with control (Figure 10e–g). In *myc2* mutant lines, antioxidant levels were significantly decreased, but nano‐Se20 partially restored their activity (Figure 10e–g), suggesting a *SlMYC2*‐independent mechanism for antioxidant accumulation.

**Figure 11:**
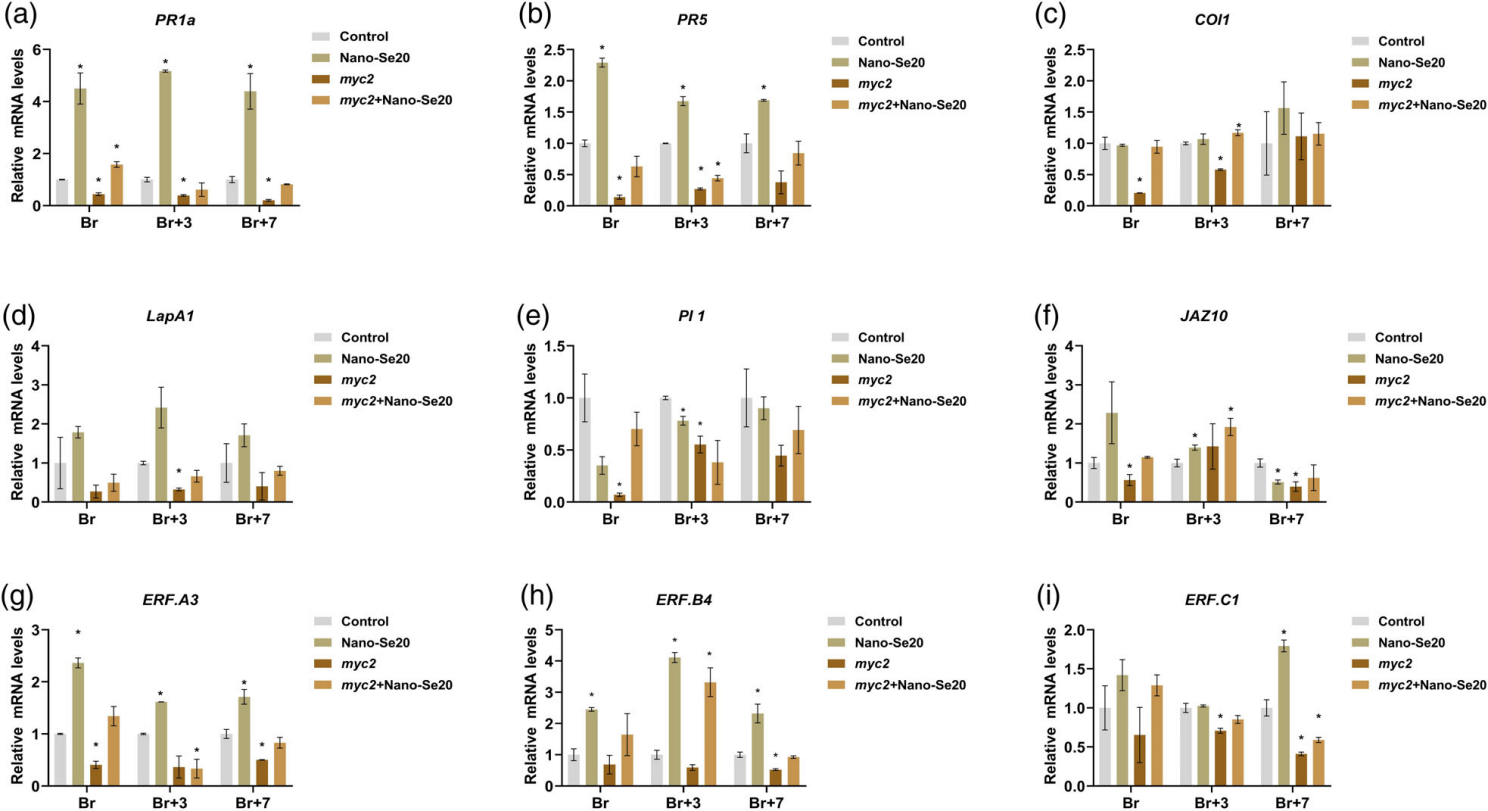
Relative expression of SA, JA, and ET‐mediated defense‐related genes after *Botrytis cinerea* infection in control, nano‐Se20, *myc2*, *myc2+*nano‐Se20 fruits. Br (Breaker), Br+3 (3 days post‐breaker) and Br+7 stage (7 days post‐breaker). (a, b) SA‐mediated defense‐related genes, *PR1a, PR5*. (c–f) JA‐mediated defense‐related genes, *COI 1, LapA1, PI 1, JAZ10*. (g–i) ET‐mediated defense‐related genes, *ERF.A3, ERF.B4, ERF.C1*. Each value represents the mean of three biological replicates. Data are shown as means ± standard deviation (SD). Asterisks indicate statistical significance using Student's *t*‐test; *P* < 0.05.

We next analyzed the expression level of SA, JA, and ET resistance‐related genes by qPCR. Nano‐Se20 treatment significantly upregulated the SA‐dependent genes *PR1a* and *PR5* at all ripening stages in WT fruits. In *myc2* mutants, these genes were downregulated but restored to near‐normal levels after nano‐Se20 treatment (Figure 11a,b). JA pathway genes (*COI1*, *LapA1*, *PI 1*, and *JAZ10*) showed no significant response to nano‐Se20 in WT fruits (Figure 11c–f), but were notably downregulated, particularly at the Br and Br+3 stages. Interestingly, in *myc2* mutants, nano‐Se20 partially restored their expression (Figure 11c–f). Additionally, ET response factors (*ERF.A1*, *ERF.A3*, *ERF.B4*, and *ERF.C1*) were significantly upregulated in response to both *B. cinerea* infection and nano‐Se20 in both the WT and *myc2* mutants (Figure 11g–i).

Collectively, these findings demonstrate that nano‐Se20 enhances tomato fruit resistance to *B. cinerea* through a multifaceted mechanism involving SA and ET signaling pathways. The ability of nano‐Se20 to restore defense gene expression and antioxidant capacity in *myc2* mutants underscores its potential as a post‐harvest treatment to improve disease resistance via *SlMYC2*‐independent pathways.

## DISCUSSION

Fruits are a vital part of the human diet, serving as key sources of essential vitamins, minerals, and antioxidants (Slavin & Lloyd, 2012). However, post‐harvest fruit loss remains a major challenge, especially for climacteric fruits like tomato, which are highly susceptible to rapid softening and decay. One of the most prevalent post‐harvest pathogens is *B. cinerea*, a filamentous fungus responsible for *B. cinerea* disease. Its infection not only accelerates fruit decay but also contributes to substantial economic losses throughout the supple chain (Zhang et al., 2013). Current chemical fungicides, such as methopyrimidine, acetone, and cyclohexylamine, are widely used to control *B. cinerea*. However, these agents raise concerns due to potential health risks, environmental impact, and the increasing emergence of post‐harvest disease resistance; without compromising fruit quality is of great importance.

In this study, we identified 20 mg L^−1^ of nano‐Se (nano‐Se20) as an optimal concentration that significantly improves tomato fruit quality. Nano‐Se20 treated fruits exhibited enhanced firmness, reduced water loss, and prolonged shelf life. Importantly, this treatment also conferred resistance to *B. cinerea* infection, making it a promising candidate for post‐harvest preservation. Transcriptional and physiological analyses revealed that nano‐Se20 orchestrates a multifaceted regulatory network during tomato ripening. The nano‐Se20 treatment upregulated key genes involved in ET biosynthesis and signaling, carotenoid and flavonoid pathways, and soluble sugar accumulation, collectively contributing to improved nutritional quality and flavor. Simultaneously, genes regulating wax and cutin biosynthesis were upregulated in enhancing physical barriers against pathogen invasion. Furthermore, nano‐Se20 modulated multiple hormone pathways associated with defense responses. In particular, genes involved in the SA and ET signaling pathways were strongly induced in response to *B. cinerea* infection. Interestingly, even in *myc2* knockout mutants, which are compromised in JA‐mediated defense, nano‐Se20 partially restored resistance to *B. cinerea* and antioxidant enzyme activity, indicating that its protective effects are partially independent of the JA‐SlMYC2 pathway. In summary, our findings demonstrate that nano‐Se20 is a potent modulator of both fruit ripening and disease resistance. By simultaneously enhancing fruit quality and activating multilayered defense networks, nano‐Se offers a promising, eco‐friendly strategy for post‐harvest management of tomato and potentially other climacteric fruits, especially for environmentally sensitive applications such as organic farming. Its dual functionality in promoting ripening while extending shelf life highlights its potential application in the fruit industry as a novel post‐harvest treatment with high commercial value. Notably, the successful application of nano‐Se in quality improvement of strawberry (Dong et al., 2023), cucumber (Jia et al., 2024), pepper (Li et al., 2020), orange plants (Gao & Tuda, 2024), peach (Ge et al., 2023), and mustard (Sarkar & Kalita, 2022), coupled with its superior advantages in cost‐effectiveness, operational convenience, and treatment stability compared with conventional preservation technologies, highlights its broad application potential in post‐harvest management of horticultural produce.

Although nano‐Se has been reported to improve the quality of horticultural crops such as pepper, tomato, and tea, its potential to extend post‐harvest shelf life remains largely unexplored. Gray mold, caused by *B. cinerea*, continues to be a major post‐harvest threat to tomatoes and many other fruits and vegetables. It also remains uncertain whether nano‐Se treatment is effective against a broader spectrum of fungal pathogens. With the rapid advancement of gene editing technologies, an increasing number of functional genes associated with ripening, defense, and quality traits are being identified. Future studies will aim to elucidate how nano‐Se influences gene function, modulates phytohormone biosynthesis pathways, and alters the molecular mechanisms of nutrient accumulation. Moreover, we will investigate the integration of nano‐Se with diverse agronomic strategies to enhance post‐harvest freshness in tomato and other climacteric fruits. Particular attention will be given to potential synergistic effects between nano‐Se treatment and other emerging preservation techniques, which may pave the way for more sustainable and effective post‐harvest management practices. While nanotechnology offers a promising and generally safe approach for agricultural applications, and nano‐Se has proven effective in enhancing tomato quality, further in‐depth research on nano‐enabled food systems is warranted to ensure comprehensive safety evaluation (Amini et al., 2014; Manoj, 2024; Sahoo et al., 2022).

## MATERIALS AND METHODS

### Sources of nano‐Se and application of nano‐Se

Nanoselenium (1500 mg L^−1^) was provided by Prof. Canping Pan, and the nano‐Se suspension (1500 mg L^−1^) used in this study was commercially obtained from Guilin JIQI Group Co. Ltd (Guilin, China). As previously characterized, the nanoparticles display well‐dispersed spherical morphology with diameters of 50–78 nm, as confirmed by transmission electron microscopy (TEM) analysis (Li et al., 2020; Liu et al., 2023). The sprayed nano‐Se solution was configured according to the concentrations of 10, 20, 30, and 40 mg L^−1^. Start foliar spraying when the plant enters stable vegetative growth (about 3 weeks), and spray once a week. After there are small fruits after flowering, foliar spraying and fruit spraying should be carried out together, and fruits of uniform size should be selected. Mark and remove excess flowers and fruits. Water was used as a control.

### Determination of Se content

Selenium standards were obtained from https://www.aladdin‐e.com/ (Aladdin, Shanghai, China, 7782‐49‐2), and selenium content was determined using a spectrophotometric method, and the absorbance was measured at 334 nm to plot the selenium standard curve. After drying in the oven, measure and record the weight of each group, and then put them back into the drying oven for drying. Dry several times to constant weight and record the data. Dry it again, take it out, and crush it into fine powder through a 180 μm (80 mesh) sieve for later use. Pipette 0.50 mL of 1000 μg mL^−1^ selenium standard stock solution and prepare a concentrated solution. The concentration is a 5 μg mL^−1^ standard solution. Heat and digest the dry fruit powder in HNO_3_: HClO_4_ = 4:1 digestion solution until the solution turns yellow. Aspirate the digested solution, place it in a stoppered Erlenmeyer flask, add 10% of the digested solution volume of 5% EDTA‐2Na solution, adjust the pH value to 1.70 with 1 mol L^−1^ HCl and 1 mol L^−1^ NaOH solution, and remove rinse with ionized water, add 20% of the digestion solution volume and 1% o‐phenylenediamine solution, mix well and place in a 50°C water bath for 40 min, rinse with distilled water 3–4 times, add toluene, layer and discard the bottom part water layer, pour the toluene layer into a quartz cuvette, and measure its absorbance at 334 nm (Huang, Tang, et al., 2023).

### Plant culture environment and transgenesis

Micro‐Tom (MT) were cultured in a greenhouse with 16 h of light and 8 h of darkness, a temperature of 22°C and 60% air humidity. The fruits of WT and 10 , 20 , 30 , and 40 mg L^−1^ treatment groups were collected for quality index and *B. cinerea* inoculation experiments. MG: fruit green ripening period, Br: fruit color breaking period, Br+1‐15 is the number of days after color breaking. The onset of ripeness is the time from flowering to color break, and markings were performed at the flowering stage and fruit color break.

### Determination of color

Fruit color was determined at different stages of ripening. A Konica Minolta chromameter (CR‐400) was used for the color measurement. The determination method was referred to Liu et al. (2014).

### Determination of Brix

Brix was measured at the Br+7 stage, and the method of determination was based on Wang et al. (2022).

### Determination of total flavonoids

The powder of fruits in liquid nitrogen quick‐frozen treatment groups of 10 , 20 , 30 , 40 mg L^−1^, and control were weighed, extracted using 80% methanol, and the absorbance values were recorded at 550 nm to calculate the relative total flavonoid content. The detection instrument is from BioTek Synergy H1.

### Determination of chlorophyll

After removing the dust from the leaf surface with a brush and blotting the surface water of leaves and fruits using paper towels, they were put into 5.0 ml EP tubes, while 3.0 ml of anhydrous ethanol: acetone mixture (V:V = 2:1) was added and placed in a dark place at room temperature and soaked overnight, during which the material was shaken several times until it turned completely white. Chlorophyll extract was used as the sample solution, the anhydrous ethanol: acetone mixture was used as the blank control, the transmittance was adjusted to 100%; and the absorbance values of the sample solution at OD_663_ and OD_645_ were measured using the detection instrument (BioTek Synergy H1). The chlorophyll content was calculated using the formula: Ca = 12.7 OD_663_ − 2.69 OD_645_ (mg L^−1^), Cb = 22.9 OD_645_ − 4.68 OD_663_ (mg L^−1^), Ca + b = 8.02 OD_645_ + 20.21 OD_663_ (mg L^−1^). C: chlorophyll concentration mg L^−1^; V: extraction volume.

### Firmness and water loss

We collected fruit from the Br+7 period, Br+10 period, and Br+15 period from the control and 20 mg L^−1^ nano‐Se treatment groups for hardness determination, TA.XTC‐18 texture analyzer (Bosin Tech, Shanghai, China). Br+7 period for the control and 20 mg L^−1^ nano‐Se treatments. The fruit were then kept in incubators with added light (22°C, 16 h light period) and weighed immediately after harvest and once a week until 25 days. The water loss rate was calculated as the ratio of fruit weight loss to initial fruit weight.

### Determination of GABA content

The extraction and detection procedures were adapted from the method described by Wang et al. (2024) with modifications. Briefly, samples were ground into powder in liquid nitrogen, and 0.1 g of frozen powder was homogenized in 1 ml of pre‐cooled 80% methanol, followed by vortexing and ice‐bath ultrasonication for 1 h. A 2 mL aliquot was centrifuged at 10 000 ***g*** for 10 min at 4°C. Then, 1 mL of the supernatant or standard solution was derivatized by adding 0.5 mL phenyl isothiocyanate–acetonitrile solution and 0.5 mL triethylamine–acetonitrile solution, followed by incubation at room temperature for 1 h. The reaction was terminated with 0.1 ml of 20% acetic acid. The derivatized solution was mixed with 2 mL *n*‐hexane, vortexed for 1 min, and allowed to separate. The lower phase was filtered twice through a 0.45 μm organic membrane into an injection vial. GABA content was analyzed via LC–MS/MS (AB Sciex QTRAP 6500) and quantified based on standard curves.

### Pathogen inoculation

Tomato fruits of Br, Br+3, and Br+7 periods were collected for surface disinfection with 75% ethanol (v/v) and washed with distilled water. Inoculation was performed as in the method of Pei et al. (2019). Use a circular punch to divide the *B. cinerea* and culture medium into blocks. Then use a dissecting needle to puncture the pericarp of the tomato fruit and attach the block to the tomato fruit. Keep in a moist growth background at 20°C and remove the *B. cinerea* block after 1 day of inoculation. Photographs were taken, and disease spot diameters were counted 72 h after inoculation.

### ET and 1‐MCP treatment

For exogenous ET treatment, mature green (MG) stage fruits were divided into three treatment groups and three control groups (with ≥10 fruits per group) and placed in sealed containers. The treatment groups were exposed to 100 ppm exogenous ET for 10 h. For 1‐MCP treatment, breaker (Br) stage fruits were similarly divided into three treatment and three control groups in sealed containers. The treatment groups were then subjected to 40 mg L^−1^ 1‐MCP for 16 h. Following treatments, fruit pericarps were collected, immediately flash‐frozen in liquid nitrogen, and stored at −80°C until analysis. Gene expression was analyzed by RT‐qPCR using primers listed in Table S3.

### Activity of antioxidant enzymes

Enzymatic activities of Superoxide Dismutase (SOD), Peroxidase (POD), and Catalase (CAT) were measured using the relevant assay kits (Solarbio Beijing, China) according to the manufacturer's instructions and optical density at 560, 470, and 240 nm, respectively, using an enzyme marker BioTek Synergy H1.

### RNA‐Seq analysis

RNA sequencing (RNA‐seq) was performed to analyze the transcriptome profiles of WT and nano‐Se 20 mg L^−1^‐treated tomato fruits at the Br+7 ripening stage, with three biological replicates for each group. RNA‐seq library construction and high‐throughput sequencing were conducted by Personalbio Technologies (Shanghai, China). Libraries were sequenced on an Illumina NovaSeq 6000 platform, generating 150‐bp paired‐end reads. Clean data were filtered, and paired reads were mapped to the tomato reference genome (SL4.0, ITAG4.0 annotations) to construct transcripts using the HISAT2 tool (Kim et al., 2015). DEGs were identified using a log2 fold change threshold of ±1.2 and *P* < 0.05 (Table S2). Pathways with *P* < 0.05 were considered significantly enriched.

### RNA isolation and RT‐qPCR analysis

Total RNA was isolated from different tomato tissues using a plant RNA extraction kit (BIOFIT, Chengdu, China). Total RNA was isolated from different tomato tissues; reverse transcription and removal of genomic DNA were performed as described (Deng et al., 2022). RT‐qPCR was performed using a SYBR qPCR mixture (Bioground, Chongqing, China) on a Bio‐Rad CFX384 real‐time PCR system (Bio‐Rad, Hercules, CA, USA) and the primers used in the expression analysis are listed in Table S3. Each type of assay was performed in three biological replicates and three technical replicates. *SlActin* was used as an internal reference gene and was calculated according to Liu et al. (2014).

### Statistical analysis

All data are expressed as mean ± SD of three or more independent experiments and were subjected to Student's *t*‐test for two‐way comparisons or anova for multivariate analysis.

## AUTHOR CONTRIBUTIONS

MW, XH, and ML planned and designed the research; XH, YX, SY, ZG, and YW performed experiments. XH and SY analyzed data. XH wrote the manuscript. MW and SY reviewed and revised the manuscript.

## CONFLICT OF INTEREST

The authors declare no conflict of interest.

## Supporting information


**Table S1.** Expression levels of genes between the control (WT) and nanoSe‐20 (NanoS20) in tomato fruits.
**Table S2.** Expression levels of DEGs between the control (WT) and nanoSe‐20 (NanoS20) in tomato fruits.
**Table S3.** List of primers used in this study.

## Data Availability

All relevant data and figures in this study can be found within the article and its Supporting Information S1. The raw transcriptome data were uploaded to the database https://ngdc.cncb.ac.cn/ with the accession number CRA020587 and are publicly accessible.
